# Physical Activity, Fatigue, and Sleep in People with Parkinson's Disease: A Secondary per Protocol Analysis from an Intervention Trial

**DOI:** 10.1155/2018/1517807

**Published:** 2018-09-06

**Authors:** S. Coe, M. Franssen, J. Collett, D. Boyle, A. Meaney, R. Chantry, P. Esser, H. Izadi, H. Dawes

**Affiliations:** ^1^Department of Sport, Health Sciences and Social Work, Oxford Brookes University, Headington Rd., Oxford OX3 0BP, UK; ^2^Nuffield Department of Primary Care Health Sciences, University of Oxford, Radcliffe Primary Care Building, Oxford OX2 6GG, UK; ^3^School of Engineering, Computing and Mathematics, Oxford Brookes University, Wheatley, Oxford OX33 1HX, UK

## Abstract

Symptoms of Parkinson's can result in low physical activity and poor sleep patterns which can have a detrimental effect on a person's quality of life. To date, studies looking into exercise interventions for people with Parkinson's (PwP) for symptom management are promising but inconclusive. The aim of this study is to estimate the effect of a clearly defined exercise prescription on general physical activity levels, fatigue, sleep, and quality of life in PwP. *Method*. PwP randomised into either an exercise group (29; 16 males, 13 females; mean age 67 years (7.12)) or a control handwriting group (36; 19 males; 17 females; mean age 67 years (5.88)) as part of a larger trial were included in this substudy if they had completed a 6-month weekly exercise programme (intervention group) and had complete objective physical activity data (intervention and control group). Sleep and fatigue were recorded from self-reported measures, and physical activity levels measured through the use of accelerometers worn 24 hours/day over a seven-day testing period at baseline and following the 24-week intervention. A Wilcoxon's test followed by a Mann–Whitney post hoc analysis was used, and effect sizes were calculated. *Results*. Participants showed a significant increase in time spent in sedentary and light activities during the overnight period postintervention in both exercise and handwriting groups (*p* < 0.05) with a moderate effect found for the change in sedentary and light activities in the overnight hours for both groups, over time (0.32 and 0.37-0.38, resp.). There was no impact on self-reported fatigue or sleep. *Conclusion*. The observed moderate effect on sedentary and light activities overnight could suggest an objective improvement in sleep patterns for individuals participating in both exercise and handwriting interventions. This supports the need for further studies to investigate the role of behavioural interventions for nonmotor symptoms.

## 1. Introduction

Parkinson's is a progressive neurological condition, and it is estimated that one in every 350 people in the UK are diagnosed with the condition. Although pharmaceutical interventions are the primary treatment option, exercise is becoming increasingly recognised as an effective addition to commonly used drug treatments for the control of both motor and nonmotor symptoms [1]. There is strong evidence supporting beneficial effects of exercise programs on disease progression, motor and nonmotor symptom management, and health and wellbeing in PwP [2–8]. Exercise interventions and a dose of 30 minutes or more a week of moderate to vigorous physical activity have been suggested to positively impact on the global nonmotor symptom burden including depression, apathy, fatigue, daytime sleepiness, sleep, and cognition [2–7, 9]. However, the evidence supporting a positive benefit of exercise to physical activity, fatigue, daytime sleepiness, and sleep is not strong [9].

PwP report sleep problems, daytime sleepiness, and fatigue as significant nonmotor symptoms [10–12]. When considering the mechanism of potential effects of exercise on these symptoms, there are a number of possible positive interactions of a physically active lifestyle including its impact to provide cognitive stimulation, meaningful social interactions, and improvement in healthy eating which may induce better sleep and reduce fatigue [13]. The interaction is complex and not well understood, with these factors interacting together in a formative and reflective manner [9, 13–16]. It is interesting that PwP are observed to have reduced motor symptoms in the morning following sleep, referred to as “sleep benefit” and that fatigue levels are lower in the morning after sleep [17]. Importantly, sleep-related symptoms in Parkinson's have been shown to relate to disease disability and worsening of motor symptoms [18]. In the general population, exercise is known to improve sleep and resulting fatigue [19].

Therefore, there is a benefit of understanding the sleep, exercise, fatigue interaction, and the limited evidence to date to support the impact of exercise on physical activity, fatigue, and sleep in PwP [20–23] and the underpinning mechanism. This study set out to estimate the potential effect of an exercise programme that combined aerobic and strength exercises on sleep patterns, activity levels, and fatigue in a group of PwP who had adhered to a prescribed aerobic and strength training exercise program and had at least three out of seven days of objective activity data collected pre- and postintervention. Therefore, the aim of this study is to estimate the potential effect of a clearly defined exercise prescription compared to an active control condition of a handwriting intervention matched for social contact on objective measures of physical activity levels and self-reported measures of fatigue, quality of life, and sleep in PwP.

## 2. Methods

### 2.1. Design

This research was carried out on a subset of data collected as part of an interventional study of exercise training in PwP in which data for PwP were obtained from a two-arm parallel single-blind phase II randomised controlled trial (RCT) of community-delivered exercise for PwP [8]. The current study included all participants from the RCT who were randomised, who adhered to the exercise group in the RCT (≥ one session a week), and who provided comprehensive objective physical activity data in the exercise group and in the handwriting group. The trial was registered with ClinicalTrials.Gov (NCT01439022).

### 2.2. Setting

Parkinson's assessments were carried out at the Movement Science Laboratory, Oxford Brookes University, Oxford, UK, the intervention took place at community leisure facilities throughout Oxfordshire and Berkshire, and the handwriting sessions took place in the home of the participants.

### 2.3. Participants

The study received National Health Service ethical approval (NRES Committee South Central–Southampton A: 11/SC/0267) and was conducted in accordance with the declaration of Helsinki.

People with idiopathic Parkinson's were recruited from neurology clinics and GP practices in the Thames Valley, UK, and through local Parkinson's UK group meetings. Inclusion criteria for PwP were as follows: (i) diagnosis of idiopathic Parkinson's (as defined by the UK Parkinson's Disease Society Brain Bank clinical diagnostic criteria [24]) and (ii) ability to walk ≥100 meters. Exclusion criteria were as follows: (i) dementia; (ii) history of additional prior neurological condition; (iii) severe depression or psychosis or a mental state that would preclude consistent active involvement with the study over its duration; (iv) cardiac precautions that would prevent the subject from participating in the intervention; (v) any known contraindication to exercise; (vi) reduced cognitive function of any cause (Mini-Mental State Examination <23); and (vii) an orthopaedic condition that limited independent walking. Participants' medication was continued as normal and was recorded.

### 2.4. Intervention

The intervention for PwP was a prescribed exercise program consisting of sessions lasting 60 minutes or a handwriting control intervention, twice a week over a period of six months; a detailed description can be found elsewhere [8]. Demographics were recorded before and after the intervention and included fatigue using the Fatigue Severity Scale (FSS) [25] and quality of life using the EQ5D-5L [26]. Selected questions from the Unified Parkinson's Disease Rating Scale, Parts 1 and 2 subsections were recorded including the following: (1) *Over the past week, have you had trouble going to sleep at night or staying asleep through the night? Consider how rested you felt after waking up in the morning;* (2) *Over the past week, have you had trouble staying awake during the daytime?* (3) *Over the past week, do you usually have trouble turning over in bed?* Only PwP who adhered (did not discontinue intervention) to the exercise program and had at least three full days of objective activity data out of total seven days for both the pre- and postassessment were included in the training response analysis. Physical activity in PwP was measured using the wrist-worn activity monitor (GENEActiv, UK). The activity monitor was worn around the wrist for seven consecutive days following an assessment. The activity monitor consisted out of a triaxial accelerometer, sampling at 100 Hz for the duration of seven days. The data were downloaded from the device onto a computer and exported as a 60-second epoch comma delimited file. A bespoke Excel macro using adjusted activity cutoff levels derived minutes per day and relevant percentages spent sedentary, performing light, moderate, or vigorous activities [27]. The physical activity data was analysed in three ways: one 24-hour period, two 12-hour periods involving a daytime section from 08.00 to 20.00, and an overnight section from 20.00 to 08.00, and an 8-hour and a 4-hour evening section from 24.00 to 08.00 and 20.00 to 24.00, respectively. Matthews et al. [28] observed that a minimum of three days was required to accurately calculate average physical activity levels; therefore, files with less than three days of recorded data were excluded from analysis.

### 2.5. Data Analysis

Descriptive statistics were calculated for demographic characteristics. For activity data, the thresholds were based on amplitude of the single vector magnitude as assessed by the triaxial accelerometer within the periods outlined above. Data was presented as median physical activity levels over a 24-hour period. Due to nonnormality, for outcome data, a Wilcoxon's test followed by a Mann–Whitney post hoc test was used to determine the changes over time and according to two intervention regimes (exercise and handwriting). Further and based on the differences, effect sizes (*r* = *Z*/√*N*) were calculated.

## 3. Results

### 3.1. Participants

Participant flow for the PwP recruited to the RCT can be found elsewhere [8]. Between December 2011 and August 2013 105 participants were recruited, 37 people adhered to the exercise intervention, and accelerometer data were complete on 29 of these participants (16 males, 13 females; mean age 67 years (7.12)) and 36 in the control handwriting group (19 males; 17 females; mean age 67 years (5.88)) and therefore were included in this secondary analysis. Demographic information is provided in supplementary (available here). Changes in health measures can be seen in Table 1. There were no significant differences pre- and postintervention (*p* > 0.05) or between groups at each time point.

### 3.2. Accelerometer

The majority of subjects wore the accelerometer for the entire seven days of each of the testing period, and although a small minority of subjects did not achieve this target, inclusion criteria for at least three days out of each seven day period were met. Tables 2 [Table tab3]–4 show physical activity measurement results. During 20:00–08:00 hours, sedentary time increased while light activity decreased in both groups with the addition of moderate activity decreasing in the control group (*p* < 0.05; Table 3). When the overnight period was considered, from 20:00 to 24:00 and 24:00 to 08:00, again sedentary time increased in both groups with a resulting reduction in time spent in light activity in the intervention group (*p* < 0.05; Table 4). Effect sizes for the same time points showed a moderate effect in the intervention and control groups (0.32 and 0.37-0.38, resp.).

## 4. Discussion

These findings support the potential use of combined aerobic and strength exercise as a moderator of general physical activity for PwP and as a potential aid for improving sleep as evidenced by reduced activity during the nighttime hours. Importantly, there was a similar effect in the active control group suggesting that behavioural exercise interventions may benefit sleep in PwP.

To examine how participants' physical activity levels changed over the course of the intervention in greater depth, the physical activity data were broken down into 12-hour, 8-hour, and 4-hour time slots. No significant differences were observed at any physical activity level during daytime; however, during the overnight period, there was a significant increase in time spent in sedentary and a significant decrease in time spent in light activity observed in both groups. The increase in sedentary activity overnight might suggest an improvement in the participants' sleep quality, and a good quality of sleep in PwP has been linked to a greater control over motor symptoms the following morning [17]. van Gilst et al. [17] suggest that the beneficial effect of sleep on motor symptoms in Parkinson's is due to improved dopaminergic function during sleep, which increases dopamine levels in the brain, a phenomenon known as “sleep benefit.” Symptoms found in Parkinson's, such as tremors and rigidity can make the initiation and maintenance of sleep more difficult than in people without the condition, and therefore any methods or techniques to help improve sleep quality in PwP would be of use [29]. The concept of subjective sleep benefit in the absence of actual objective sleep benefit may also be of clinical relevance to PwP and has been associated with nonmotor improvements [30]. Between 24.00 and 08.00, sedentary activity increased whilst time spent in light activity decreased. Participants spent significantly more time sedentary following both interventions between 20.00 and 24.00 whilst significantly less time was also observed at the light activity level during this time period. This could suggest that PwP who took part in the exercise intervention went to sleep earlier, or were just less active in the evenings to compensate for the increased activity as a result of the intervention. The effect of this increased daytime activity could therefore have a resulting impact on sleep quantity and quality, reducing daytime tiredness and potentially leading to beneficial changes in both motor and nonmotor symptoms [31]. Engagement with cognitive and fine motor skill tasks may reduce daytime sleepiness and increase nighttime sleep and therefore the active control group may have been receiving cognitive stimulation which may have improved sleep time [32].

Interestingly, in the current study there was no impact on fatigue as shown in previous research [33]. To date, there is limited evidence to support the impact of exercise on physical activity, fatigue, or sleep in PwP [20–23]. Variations of resistance exercise training have been shown to specifically improve subjective sleep quality whilst being safe and feasible for PwP [34]. The combination of aerobic and resistance exercise interventions may be optimal for reducing fatigue, increasing physical activity, and improving sleep quality in PwP, as this was shown in healthy elderly people who often experience disturbed sleep [35]. In addition, the handwriting control group had engagement with cognitive and fine motor tasks which were also shown to improve sleep. However, mechanisms underlying improved sleeping habits differ and more robust research needs to be conducted in PwP. Demographic data were collected pre- and postintervention, and although there were no significant changes, sleep problems and daytime sleepiness (as recorded via the UPDRS subsections) tended to improve over the 6-month period and should be evaluated in a full trial.

There were some limitations to the current study in that the actual timings of sleep or sleep patterns were not recorded throughout the study, with sleep times being estimated through increased sedentary behaviour. It would be worth performing a subanalysis for those who experience sleep issues or daytime sleepiness at baseline; however, if this was performed in the current trial, the sample size would be small and therefore the findings of limited relevance. Lack of administration of more valid scales and tools (e.g., objective assessment of sleep with polysomnography and Pittsburg Sleep Quality Index) to assess sleep quality is an important limitation of this study. However, the results from this trial add to the current evidence for exercise interventions as a mean to improve symptom management and physical and psychological functioning in PwP [3–8].

## 5. Conclusion

Evidence has shown that physical activity is beneficial for PwP, yet with little research showing its potential to improve motor and nonmotor symptoms, quality of life, and sleep patterns [36]. The current study looked at the effect of an exercise intervention programme and an active handwriting control group on general physical activity levels in PwP, and it was found that participants experienced a significant increase in time spent sedentary overnight which may have been linked to an improvement in sleep. Future studies should explore the role of physical activity and behavioural interventions to determine the effect on nonmotor symptoms including sleep and fatigue in Parkinson's.

## Figures and Tables

**Table 1:** Measures of health before and after the 24-week exercise intervention.

	Exercise		Handwriting	
	Before	After	Before	After
FSS	3 ± 1.38	3 ± 1.35	4 ± 1.46	3 ± 1.56
EQ5D-5L	79.21 ± 12.22	78.48 ± 17.89	76.58 ± 16.29	74.75 ± 17.94
Sleep problems^a^	1.5 ± 1.43	1.3 ± 1.26	1.4 ± 1.33	1.5 ± 1.38
Daytime sleepiness^a^	1.5 ± 0.78	1.5 ± 0.87	1.2 ± 0.94	1.3 ± 0.91
Turning in bed^a^	0.5 ± 0.69	0.7 ± 0.65	0.7 ± 0.62	0.8 ± 0.84

Values are means ± standard deviations. A Wilcoxon signed rank test was used to determine differences between pre- and postintervention followed by a Mann–Whitney test for between-group differences, with ^*∗*^ *p* < 0.05 and ^*∗∗*^ *p* > 0.05, respectively. FSS: fatigue severity scale; ^a^from UPDRS, Unified Parkinson's Disease Rating Scale Parts 1 and 2 subscores.

**Table 2:** Objective physical activity levels of participants following a 24-week exercise intervention averaged over a 24-hour period.

	Exercise				Handwriting			
	Before	After	*Z*	ES	Before	After	*Z*	ES
SED	0.70	0.75	−0.832	0.11	0.68	0.73^*∗*^	3.488	0.41
LIGHT	0.26	0.23	−0.789	0.10	0.28	0.24^*∗*^	−3.126	0.37
MOD	0.04	0.03	−0.335	0.04	0.03	0.03^*∗*^	−2.388	0.28
VIG	0	0	−0.355	0.05	0	0^*∗*^	−2.293	0.27

The exercise intervention consisted of both aerobic and anaerobic exercise training for 60 minutes, twice a week for 24 weeks. Values are medians of fraction of time spent at each level of activity using an accelerometer. A Wilcoxon signed rank test was used to determine differences between pre- and postintervention followed by a Mann–Whitney test for between-group differences, with ^*∗*^ *p* < 0.05 and ^*∗∗*^ *p* < 0.05, respectively. Effect sizes (ES) were calculated using the equation *r* = *Z*/√*N* and were over time in each group. SED, sedentary activity level; LIGHT, light activity level; MOD, moderate activity level; VIG, vigorous activity level.

**Table 3 tab3:** Objective physical activity levels of participants following a 24-week exercise intervention averaged over two 12-hour periods.

	Exercise				Handwriting			
	Before	After	*Z*	ES	Before	After	*Z*	ES
08.00–20.00								
SED	0.65	0.63	−0.422	0.06	0.64	0.66	0.927	0.11
LIGHT	0.30	0.32	−0.638	0.08	0.31	0.27	−0.597	0.07
MOD	0.03	0.05	−0.919	0.12	0.03	0.02	−0.408	0.05
VIG	0	0	−0.118	0.02	0	0	−1.719	0.20

20.00–08.00								
SED	**0.75**	**0.87** ^*∗*^	−2.411	**0.32**	**0.69**	**0.86** ^*∗*^	3.236	**0.38**
LIGHT	**0.22**	**0.12** ^*∗*^	−2.411	**0.32**	**0.26**	**0.13** ^*∗*^	3.111	**0.37**
MOD	0.01	0.01	−1.344	0.18	0.02	0.01^*∗*^	−2.662	0.31
VIG	0	0	−1.099	0.14	0	0	−1.112	0.13

The exercise intervention consisted of both aerobic and anaerobic exercise training for 60 minutes, twice a week for 24 weeks. Values are medians of fraction of time spent at each level of activity using an accelerometer. A Wilcoxon signed rank test was used to determine differences between pre- and postintervention followed by a Mann–Whitney test for between-group differences, with ^*∗*^ *p* < 0.05 and ^*∗∗*^ *p* < 0.05, respectively. Effect sizes (ES) were calculated using the equation *r* = *Z*/√*N*. SED, sedentary activity level; LIGHT, light activity level; MOD, moderate activity level; VIG, vigorous activity level.

**Table 4:** Objective physical activity levels of participants following a 24-week exercise intervention averaged over one eight-hour overnight and one four-hour periods.

	Exercise				Handwriting			
	Before	After	Z	ES	Before	After	Z	ES
24.00–08.00								
SED	0.76	0.89^*∗*^	−2.000	0.26	0.67	^*∗*^0.89	2.906	0.34
LIGHT	0.20	0.09^*∗*^	−2.108	0.28	0.28	^*∗*^0.09	−2.828	0.33
MOD	0.01	0	−1.658	0.22	0.01	^*∗*^0	−2.080	0.25
VIG	0	0	−1.734	0.23	0	0	−1.821	0.21

20.00–24.00								
SED	0.63	0.71^*∗*^	−2.389	0.31	0.68	^*∗*^0.73	2.121	0.25
LIGHT	0.24	0.18^*∗*^	−2.433	0.32	0.21	0.14	−1.579	0.19
MOD	0	0.01	−1.622	0.21	0.01	0.01	−0.688	0.08
VIG	0	0	0	0	0	0	−0.307	0.04

The exercise intervention consisted of both aerobic and anaerobic exercise training for 60 minutes, twice a week for 24 weeks. Values are medians of fraction of time spent at each level of activity using an accelerometer. A Wilcoxon signed rank test was used to determine differences between pre- and postintervention followed by a Mann–Whitney test for between-group differences, with ^*∗*^ *p* < 0.05 and ^*∗∗*^ *p* < 0.05, respectively. Effect sizes (ES) were calculated using the equation *r* = *Z*/√*N*. SED, sedentary activity level; LIGHT, light activity level; MOD, moderate activity level; VIG, vigorous activity level.

## Data Availability

The data used to support the findings of this study are available from the corresponding author upon request.

## Supplementary Materials

Supplementary Materials Demographics of both the exercise and handwriting groups at baseline including disease duration and disease severity (using the Unified Parkinson's Disease Rating Scale). Values are means and standard deviations.
