# Supplementary material for: Physical Activity, Fatigue, and Sleep in People with Parkinson's Disease: A Secondary per Protocol Analysis from an Intervention Trial
Source: Parkinsons Dis. 2018 Sep 6;2018:1517807. doi: 10.1155/2018/1517807 (PMC6146558; doi:10.1155/2018/1517807)
Supplement: Supplementary Materials — Demographics of both the exercise and handwriting groups at baseline including disease duration and disease severity (using the Unified Parkinson's Disease Rating Scale). Values are means and standard deviations. [file 1517807.f1.docx]

Supplementary file: Demographics of both the exercise and handwriting group at baseline

|  | Exercise | Handwriting |
| --- | --- | --- |
| Disease duration (years) | 4.82 ± 4.05 | 5.50 ± 4.20 |
| Disease severity  UPDRS-NMS | 8.59 ± 4.45 | 7.36 ± 4.77 |
| UPDRS-III | 15.10 ± 10.04 | 18.97 ± 10.70 |

UPDRS: Unified Parkinson’s Disease Rating Scale; NMS: non motor symptoms, values are means +/- standard deviations.
